# Overweight/obesity affects histological features and inflammatory gene signature of synovial membrane of Rheumatoid Arthritis

**DOI:** 10.1038/s41598-019-46927-w

**Published:** 2019-07-18

**Authors:** Stefano Alivernini, Barbara Tolusso, Maria Rita Gigante, Luca Petricca, Laura Bui, Anna Laura Fedele, Clara Di Mario, Roberta Benvenuto, Francesco Federico, Gianfranco Ferraccioli, Elisa Gremese

**Affiliations:** 1 Division of Rheumatology, Fondazione Policlinico Universitario A. Gemelli IRCCS, Rome, Italy; 2 Institute of Rheumatology, Università Cattolica del Sacro Cuore, Rome, Italy; 3 Institute of Pathology, Fondazione Policlinico Universitario A. Gemelli IRCCS, Rome, Italy; 4 Institute of Pathology, Università Cattolica del Sacro Cuore, Rome, Italy

**Keywords:** Rheumatoid arthritis, Translational research

## Abstract

Overweight/obesity influence disease burden and clinical outcome of Rheumatoid Arthritis (RA). The impact of overweight/obesity on synovial tissue (ST) inflammation is largely unknown. Here, we investigated the histological and transcriptional signature of ST obtained from RA in different disease phases (disease onset, failure to first-line conventional DMARDs and in sustained clinical and ultrasound remission) finding that overweight/obese DMARDs naive RA showed higher likelihood of follicular synovitis, higher IHC scores for sublining inflammatory cells (CD68^+^, CD21^+^ and CD20^+^) and higher IL-1RA plasma levels than normal weight RA. Regardless to the synovitis pattern, overweight/obese DMARDs naive RA showed a worse clinical response to “Treat-to-target” (T2T) than normal weight RA at 6 and 12 months follow-up. Conversely, MTX-IR RA did not show significant differences in synovial inflammation based on BMI category. Overweight/obese RA in stable clinical and US remission showed higher degree of residual synovitis in terms of sublining CD68^+^, CD20^+^ cells and lining and sublining CD3^+^ compared to normal weight RA. Finally, gene expression profile analysis revealed that ST of overweight/obese DMARDs naive RA is enriched by CCL3 and MyD88 compared to normal weight RA in sustained disease remission, the latter correlating with BMI and IHC scores for synovial CD68^+^ cells. These findings suggest that indeed overweight/obese RA show higher degree of synovitis at disease onset and after remission achievement that influences the response rate to T2T and should be considered within the management of patients with RA.

## Introduction

There are increasing evidences that overweight and obesity are risk factors for the development of Rheumatoid Arthritis (RA) and that a high Body Mass Index (BMI) is associated with high disease activity and disability at disease onset^1–3^, being an independent factor of worse clinical response to RA treatment^4–6^. Despite this, pooling data derived from multiple imaging studies showed that BMI is inversely associated with joint inflammation compared to normal weight RA^7–9^.

A recent study on animal models of arthritis confirmed that the effect of obesity on synovial tissue (ST) inflammation is contingent on the disease phase being more significant at disease onset and in the resolution phase^10^, as promoted by the aberrant release of pro-inflammatory cytokines from adipocytes in obese arthritic animals which enhance the persistence of pro-inflammatory cells within the ST delaying the inflammatory process resolution. Despite this, it has to be mentioned that obese RA were found to be protected by bone damage^11,12^. To date, no information is available on ST analysis of such population in humans, with a lack of knowledge about the molecular mechanisms associated with synovial inflammation in overweight/obese RA, in different disease phases, regulating tissue inflammation and bone protection.

Based on this, the aims of the present study were: (i) to define the histological features of RA ST in different disease phases (disease onset or first conventional DMARD failure) in terms of CD68^+^, CD21^+^, CD3^+^ and CD20^+^ cells ST distribution based on the BMI category; (ii) to dissect if overweight/obesity status is associated with the aberrant expression of cytokines related to inflammation [i.e. Interleukin-6 (IL-6)] and bone damage [i.e. Interleukin-1 Receptor Antagonist (IL-1RA)] in naive to treatment RA; (iii) to define if BMI category, associated with ST characteristics, may influence the response rate to a treat to target strategy in naive to treatment RA and (iv) to dissect weather the BMI category may affect the histological features of residual synovitis in RA in stable clinical and ultrasound remission.

## Results

### Demographic, clinical and immunological characteristics of the study cohorts

Table 1 summarizes the demographic, clinical and immunological characteristics of the study cohorts. In particular, naive to treatment RA were significantly younger than MTX-IR RA (p = 0.03) and RA in stable clinical and ultrasound remission (p = 0.04). Naive to treatment RA had similar disease activity than MTX-IR RA (3.7 ± 1.3 vs 3.3 ± 1.1; p = 0.10) and significantly higher than RA in stable clinical and ultrasound remission (1.0 ± 0.4; p < 0.001). Considering the BMI categories, the overweight/obesity rate was comparable among the three study cohorts (60.0% naive RA had BMI ≥ 25 kg/m^2^ compared to 58.1% MTX-IR RA and 60.0% RA in sustained clinical and ultrasound remission; p > 0.05). Considering the autoimmune profile, there was no significant difference in terms of autoantibody positivity in the three study cohorts based on the BMI category (p = 0.52, p = 0.48 and p = 0.61 comparing autoantibody positivity based on BMI category within the naive to treatment RA, MTX-IR RA and RA in sustained remission cohorts respectively). Considering the bone damage, the percentage of erosive naive RA at disease onset was 37.1% with no significant difference stratifying according to the BMI category (28.6% in RA patients with BMI < 25 kg/m^2^ and 42.9% in patients with BMI ≥ 25 kg/m^2^ respectively; p = 0.17). Moreover, demographic, clinical and immunological characteristics of the study cohorts stratified based on BMI category are summarized in Supplementary Table 1.

**Table 1:** Demographic, clinical and immunological characteristics of the study cohorts.

	Naive RA (n = 70)	MTX-IR RA (n = 43)	Remission RA (n = 25)	*p* ^*a*^	*p* ^*b*^	*p* ^*c*^
Female, n(%)	55 (78.6)	39 (90.7)	21 (84.0)	0.10	0.39	0.40
Age, years (mean ± SD)	53.5 ± 15.4	59.5 ± 13.8	57.2 ± 14.9	**0.03**	**0.04**	0.65
Disease duration, years (mean ± SD)	1.9 ± 0.7	6.1 ± 4.6	9.7 ± 2.8	**<0.001**	**<0.001**	**0.01**
AB positivity, n(%)	36 (51.4)	25 (58.1)	17 (68.0)	0.48	0.11	0.42
DAS, (mean ± SD)	3.7 ± 1.3	3.3 ± 1.1	1.1 ± 0.4	0.10	**<0.001**	**<0.001**
BMI ≥ 25 Kg/m^2^, n(%)	42 (60.0)	25 (58.1)	15 (60.0)	0.54	0.59	0.00
BMI, (mean ± SD)	27.2 ± 5.8	26.7 ± 5.3	25.8 ± 4.1	0.64	0.44	0.34
ESR, mm/1^st^ hour (mean ± SD)	53.3 ± 30.5	48.4 ± 32.6	17.0 ± 16.1	0.43	**<0.001**	**<0.001**
CRP, mg/L (mean ± SD)	22.1 ± 22.1	18.4 ± 22.2	2.1 ± 2.0	0.27	**<0.001**	**<0.001**
**Treatment regimen**						
MTX dose, mg/week	—	13.8 ± 5.7	14.4 ± 3.6	—	—	0.64
Etanercept 50 mg/week	—	—	13 (52.0)	—	—	—
Adalimumab 40 mg/2 weeks	—	—	12 (48.0)	—	—	—

**AB**: autoantibody; **BMI**: Body Mass Index; **CRP**: C Reactive Protein; **DAS**: Disease Activity Score; **ESR**: Erythrocyte Sedimentation Rate; **RA**: Rheumatoid Arthritis. **MTX-IR**: Methotrexate inadequate responder; p^a^: naïve RA vs MTX-IR RA patients; p^b^: naïve RA vs remission RA patients; p^c^: MTX-IR RA vs remission RA patients; **SD**: Standard Deviation; **Bold**: p < 0.05.

### BMI influences IHC characteristics of RA at disease onset in terms of synovial resident inflammatory cells

**Figure 1:**
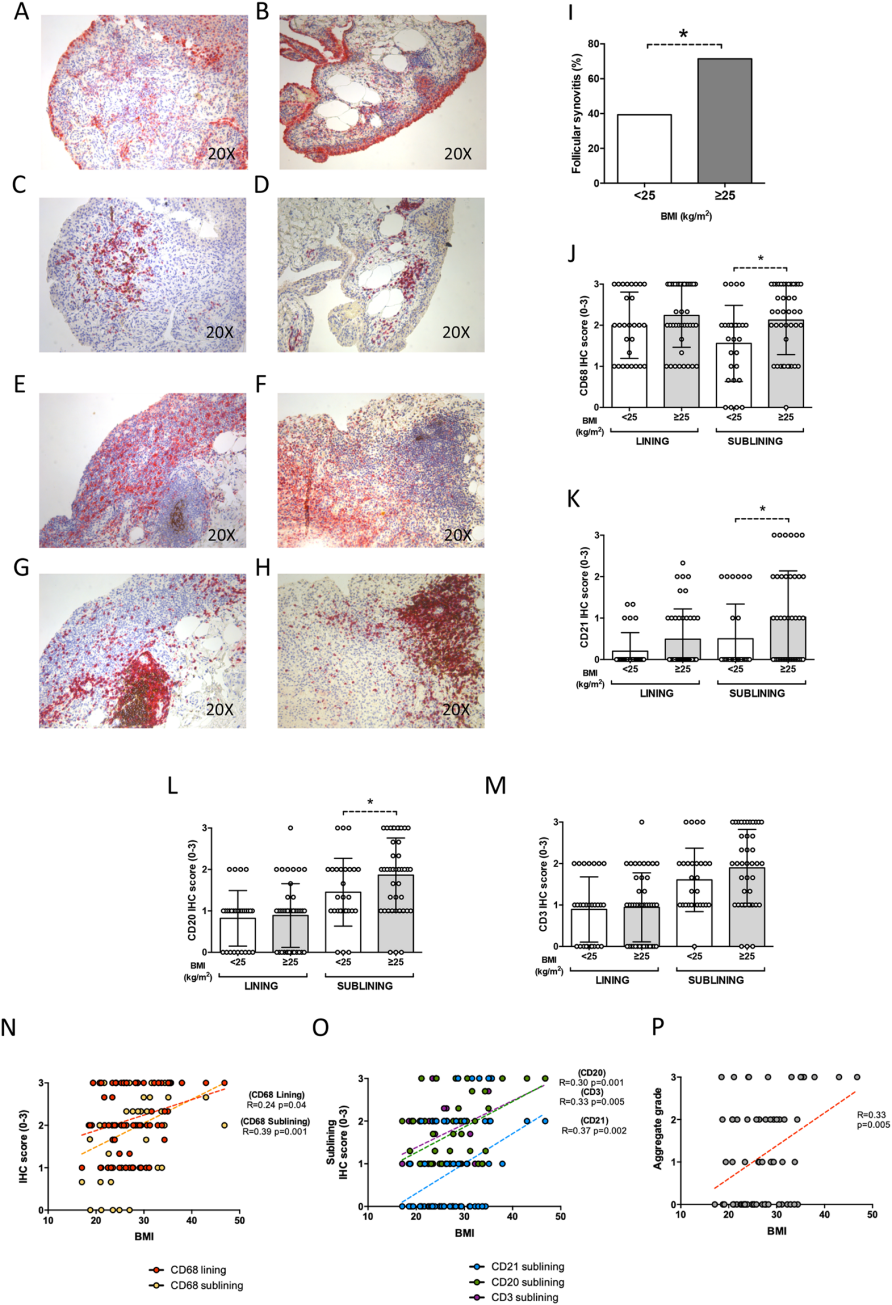
(**A**–**P**) Legend: IHC staining for CD68/CD21 and CD3/CD20 on ST of naive to DMARDs treatment Rheumatoid Arthritis patients based on the BMI category. (**A**,**B**) Example photos of CD68(red)/CD21(brown) staining of ST biopsies from naive to DMARDs RA with BMI < 25 kg/m^2^; (**C**,**D**) Example photos of CD3(red)/CD20(brown) staining of ST biopsies from naive to DMARDs RA with BMI < 25 kg/m^2^; (**E**,**F**) Example photos of CD68(red)/CD21(brown) staining of ST biopsies from naive to DMARDs RA with BMI ≥ 25 kg/m^2^; (**G**,**H**) Example photos of CD3(red)/CD20(brown) staining of ST biopsies from naive to DMARDs RA patients with BMI ≥ 25 kg/m^2^ (all magnifications 20X); (**I**) Rate of follicular synovitis in naive to DMARDs RA at disease onset with BMI < 25Kg/m^2^ (39.3%) vs RA with BMI ≥ 25 kg/m^2^ (71.4%, *p = 0.001); (**J**) Lining and sublining IHC score for CD68 cells in ST of naive to DMARDs RA based on BMI category; Lining and sublining CD68 IHC score of overweight/obese vs normal weight naive to DMARDs RA (p = 0.21 and *p = 0.01 respectively); (**K**) Lining and sublining IHC score for CD21 cells in ST of naive to DMARDs RA based on BMI category; Lining and sublining CD21 IHC score of overweight/obese vs normal weight naive to DMARDs RA (p = 0.06 and *p = 0.03 respectively); (**L**) Lining and sublining IHC score for CD20 cells in ST of naive to DMARDs RA based on BMI category; Lining and sublining CD20 IHC score of overweight/obese vs normal weight naive to DMARDs RA (p = 0.71 and *p = 0.05 respectively); (**M**) Lining and sublining IHC score for CD3 cells in ST of naive to DMARDs RA based on BMI category; Lining and sublining CD3 IHC score of overweight/obese vs normal weight naive to DMARDs RA patients (p = 0.79 and p = 0.17 respectively); (**N**) Correlations between lining and sublining IHC scores for CD68^+^ cells and BMI in naive to DMARDs RA patients; (**O**) Correlations between sublining IHC score of CD21^+^, CD20^+^ and CD3^+^ cells and BMI in naive to DMARDs RA patients; (**P**) Correlations between synovial aggregate grade value and BMI (R = 0.33; p = 0.005) in naïve to DMARDs RA; RA: Rheumatoid Arthritis; DMARDs: Disease Modifying Anti-Rheumatic Drugs; BMI: Body Mass Index; ST: Synovial Tissue; IHC: Immunohistochemistry; CD: Cluster Designation.

Among naive to treatment RA, subjects with BMI ≥ 25 Kg/m^2^ showed a higher rate of synovial inflammation compared to normal weight RA (Fig. 1A–H). In particular, naïve RA with BMI ≥ 25 Kg/m^2^ had more likely follicular synovitis pattern (71.4%) than normal weight naïve RA (39.3%; p = 0.001) (Fig. 1I). Double immunohistochemistry showed that naïve RA with BMI ≥ 25 Kg/m^2^ showed significantly higher IHC score for sublining CD68^+^ (2.1 ± 0.8) (Fig. 1J), CD21^+^ (1.0 ± 1.1) (Fig. 1K) and CD20^+^ cells (1.9 ± 0.9) (Fig. 1L) compared to normal weight RA (1.5 ± 0.9 for CD68^+^ cells; p = 0.01; 0.5 ± 0.8 for CD21^+^ cells; p = 0.03; and 1.4 ± 0.8 for CD20^+^ cells; p = 0.05, respectively). Moreover, considering the whole cohort of naive RA, BMI value directly correlated with the IHC scores for lining CD68^+^ (R = 0.24; p = 0.04), lining CD21^+^ (R = 0.36; p = 0.002) and sublining CD68^+^ (R = 0.39; p = 0.001) (Fig. 1N), sublining CD21^+^ (R = 0.37; p = 0.002), sublining CD20^+^ (R = 0.38; p = 0.001) and sublining CD3^+^ cells (R = 0.33; p = 0.005) respectively (Fig. 1O) and with the synovial aggregate grade (R = 0.33; p = 0.005) (Fig. 1P).

### Overweight/obese RA patients at disease onset show aberrant levels of inflammatory cytokines associated to the degree of synovial inflammation

To assess if overweight/obesity status might be related to aberrant systemic inflammation, levels of inflammatory (i.e. IL-6) and anti-inflammatory (i.e. IL-1RA) cytokines were assessed in PB of naive RA and in RA patients in stable clinical and ultrasound remission at the time of ST biopsy. Plasma levels of IL-6 were significantly higher at RA onset (48.4 ± 56.5 pg/ml) than in RA in sustained remission (3.4 ± 7.9 pg/ml; p < 0.001), whereas IL-1RA plasma levels were not statistically different between naïve RA (647.3 ± 397.9 pg/ml) and RA in sustained remission (464.4 ± 217.4 pg/ml; p = 0.07).

**Figure 2:**
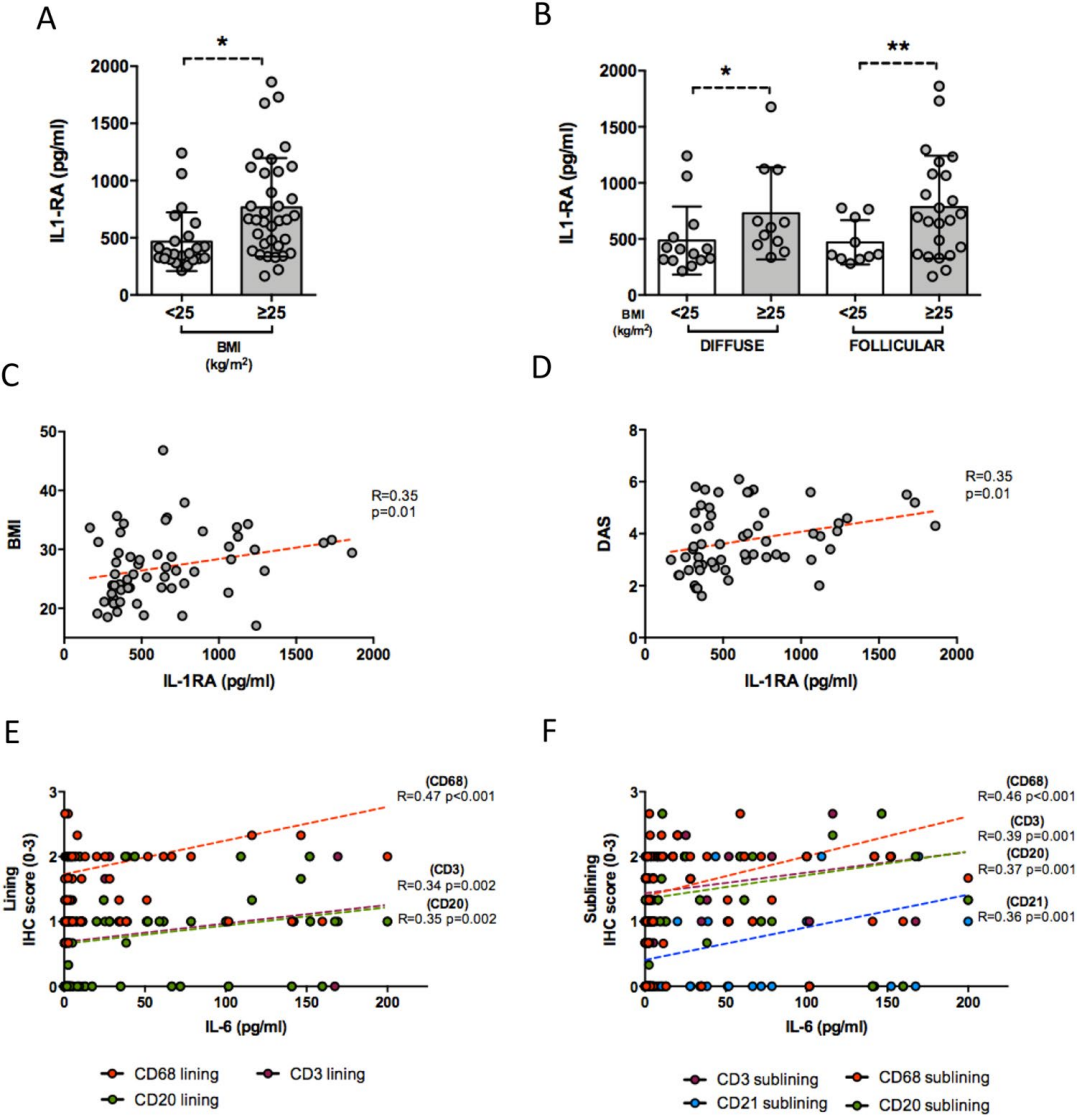
(**A**–**F**) Legend: Association between cytokines plasma levels, BMI and degree of synovial inflammation in naïve RA patients and in RA patients in stable clinical and ultrasound remission. (**A**) IL-1RA plasma levels in naïve to treatment RA patients based on the BMI category, *p = 0.002; (B) IL-1RA plasma levels in naïve to treatment RA based on the BMI category and synovitis pattern, *p = 0.05 and **p = 0.04; (**C**) Correlation between IL-1RA plasma levels and BMI value in naïve to treatment RA patients; (**D**) Correlation between IL-1RA plasma levels and DAS value in naïve to treatment RA patients; (**E**) Correlations between IL-6 plasma levels and lining IHC scores for CD68^+^, CD3^+^ and CD20^+^ cells in naïve to treatment RA patients and RA patients in stable clinical and ultrasound remission; (**F**) Correlations between IL-6 plasma levels and sublining IHC scores for CD68^+^, CD21^+^, CD3^+^ and CD20^+^ cells in naïve to treatment RA patients and RA in stable clinical and ultrasound remission; RA: Rheumatoid Arthritis; BMI: Body Mass Index; IHC: Immunohistochemistry; CD: Cluster Designation.

Considering naive RA based on the BMI category, patients with BMI ≥ 25 Kg/m^2^ showed significantly higher IL-1RA plasma levels (766.5 ± 431.1 pg/ml) that patients with BMI < 25 Kg/m^2^ (465.9 ± 257.0; p = 0.002) (Fig. 2A). Moreover, stratifying naive RA according to the synovitis pattern, patients with BMI ≥ 25 Kg/m^2^ showed higher IL-1RA plasma levels regardless to the presence of diffuse (728.5 ± 410.5 pg/ml) or follicular pattern (784.2 ± 457.8 pg/ml) compared to patients with BMI < 25 kg/m^2^ (485.6 ± 302.9 pg/ml in RA patients with diffuse synovitis; p = 0.05; 469.3 ± 197.4 pg/ml in RA patients with follicular synovitis; p = 0.04) (Fig. 2B). Moreover, in naive RA IL-1RA plasma levels directly correlated with DAS (R = 0.35; p = 0.01) and BMI (R = 0.35; p = 0.01) suggesting a tight link between disease activity and burden of inflammation as well as fat tissue excess at RA onset (Fig. 2C,D). Considering the presence of bone damage, erosive naive RA showed higher IL-6 (66.6 ± 56.9 pg/ml) and IL-1RA plasma levels (789.5 ± 412.0 pg/ml) than no erosive patients (IL-6: 38.4 ± 54.3 pg/ml; p = 0.05 and IL-1RA: 571.1 ± 374.1 pg/ml; p = 0.04). Conversely, RA in sustained remission without erosive disease showed higher IL-1RA plasma levels (577.0 ± 221.0 pg/ml) than no erosive ones (316.9 ± 88.9 pg/ml; p = 0.002) (see Supplementary Fig. 1A,B), directly related to BMI value (see Supplementary Table 2). As a consequence, considering the presence of erosions based on BMI category, the lowest plasma IL-6/IL-1RA ratio was found in overweight/obese naive RA without erosive disease than in normal weight naive RA with or without erosive disease and overweight/obese naive RA with erosive disease (p = 0.01 for all comparisons) (see Supplementary Fig. 1C).

Considering the pooled cohorts of naive RA and RA in remission, IL-6 plasma levels directly correlated with the IHC scores for lining CD68^+^ (R = 0.47; p < 0.001), lining CD3^+^ (R = 0.34; p = 0.002) and lining CD20^+^ (R = 0.35; p = 0.002) cells (Fig. 2E) and with the IHC scores for sublining CD68^+^ (R = 0.46; p < 0.001), sublining CD21^+^ (R = 0.36; p = 0.001), sublining CD3^+^ (R = 0.39; p < 0.001) and sublining CD20^+^ (R = 0.37; p = 0.001) cells (Fig. 2F).

### BMI category influences the response rate to T2T strategy in naive to treatment RA independently from synovitis pattern

The analysis of overweight/obesity status effect on remission achievement after T2T scheme, revealed that naive RA with BMI ≥ 25 kg/m^2^ showed significantly lower rate of DAS remission after 6 months (28.2%) and 12 months (37.8%) follow-up compared to naive RA with BMI < 25 kg/m^2^ (57.1% and 67.9% RA in DAS remission after 6 and 12 months of follow-up; p = 0.02 and p = 0.01 respectively). Stratifying naive RA based on the synovitis pattern detected at the ST level, before the beginning of DMARDs treatment, RA with BMI ≥ 25 kg/m^2^ and follicular synovitis showed significantly lower rate of DAS remission achievement after 6 (34.5%) and 12 months (37.8%) follow-up compared to RA with BMI < 25 kg/m^2^ and diffuse synovitis [64.7% and 70.6% RA with BMI < 25 kg/m^2^ and diffuse synovitis in DAS remission after 6 (p = 0.005) and 12 months (p = 0.03) follow-up] treated with the T2T (see Supplementary Fig. 2A,B). Moreover, considering the IHC scores for inflammatory synovial cells, there were no significant differences in terms of baseline IHC scores for CD68^+^, CD21^+^, CD20^+^ and CD3^+^ cells comparing RA reaching or not DAS remission after 6 and 12 months follow-up, regardless to the BMI category (data not shown). None of the naive RA experienced significant weight variation leading to change of the initial BMI category during the follow-up.

### BMI does not influence IHC characteristics of RA patients inadequately responding to MTX

**Figure 3:**
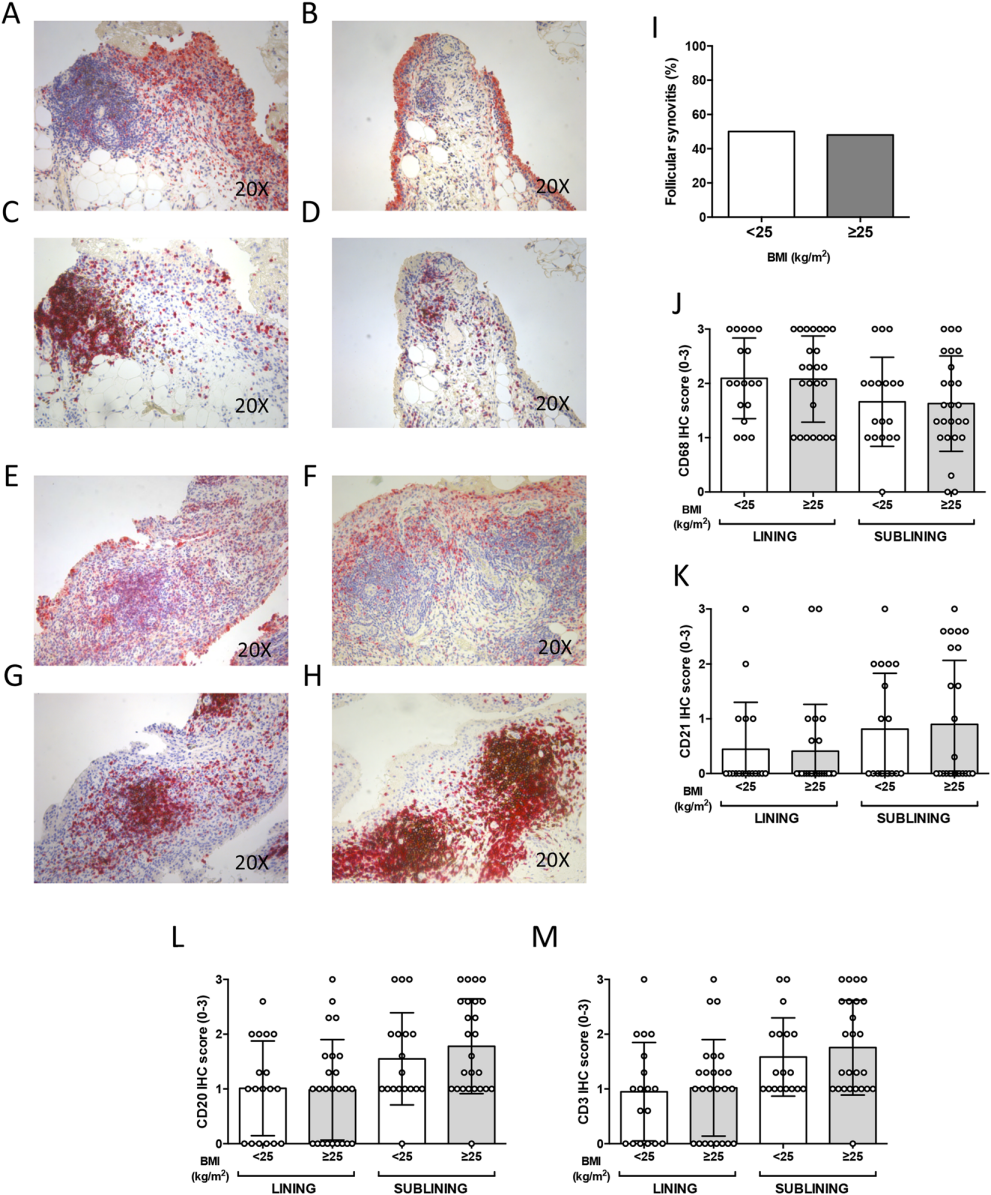
(**A**–**M**) Legend: IHC staining for CD68/CD21 and CD3/CD20 on ST of patients with Rheumatoid Arthritis inadequately responder to MTX based on the BMI category. (**A**,**B**) Example photos of CD68(red)/CD21(brown) staining of ST biopsies from MTX-IR RA with BMI < 25 kg/m^2^; (**C**,**D**) Example photos of CD3(red)/CD20(brown) staining of ST biopsies from MTX-IR RA with BMI < 25 kg/m^2^; (**E**,**F**) Example photos of CD68(red)/CD21(brown) staining of ST biopsies from MTX-IR RA with BMI ≥ 25 kg/m^2^; (**G**,**H**) Example photos of CD3(red)/CD20(brown) staining of ST biopsies from MTX-IR RA patients with BMI ≥ 25 kg/m^2^; (all magnifications 20X) (**I**) Rate of follicular synovitis in MTX-IR RA with BMI < 25 Kg/m^2^ (50.0%) vs MTX-IR RA with BMI ≥ 25 Kg/m^2^ (48.0%; p = 0.97); (**J**) Lining and sublining IHC score for CD68 cells in ST of MTX-IR RA based on BMI category; Lining and sublining CD68 IHC score of overweight/obese vs normal weight MTX-IR RA (p = 0.95 and p = 0.90 respectively); (**K**) Lining and sublining IHC score for CD21 cells in ST of MTX-IR RA based on BMI category; Lining and sublining CD21 IHC score of overweight/obese vs normal weight MTX-IR RA (p = 0.89 and p = 0.79 respectively); (**L**) Lining and sublining IHC score for CD20 cells in ST of MTX-IR RA based on BMI category; Lining and sublining CD20 IHC score of overweight/obese vs normal weight MTX-IR RA (p = 0.92 and p = 0.38 respectively); (**M**) Lining and sublining IHC score for CD3 cells in ST of MTX-IR RA based on BMI category; Lining and sublining CD3 IHC score of overweight/obese vs normal weight MTX-IR RA (p = 0.80 and p = 0.49 respectively); RA: Rheumatoid Arthritis; MTX-IR: inadequately responder to Methotrexate; ST: Synovial Tissue; IHC: Immunohistochemistry; BMI: Body Mass Index; CD: Cluster Designation.

Since overweight/obesity status affected the response rate to T2T strategy (see Supplementary Fig. 2A,B), we analysed histological synovial features of an independent cohort of MTX-IR RA, finding no significant difference in terms of ST inflammation stratifying patients based on the BMI category (Fig. 3A–H). In particular, in MTX-IR RA there was a similar rate of follicular synovitis comparing patients with BMI ≥ 25 kg/m^2^ (48.2%) and patients with BMI < 25 kg/m^2^ (52.1%; p = 0.83) (Fig. 3I). Moreover, there were no significant differences in lining and sublining IHC scores for CD68^+^, CD21^+^, CD3^+^ and CD20^+^ cells in MTX-IR RA based on the BMI category (Fig. 3J,M). Therefore, the inflammation driving the MTX-IR seems to reside in the joint not in the fat tissue.

### BMI influences IHC characteristics of synovial tissue residual inflammation of RA patients in stable clinical and ultrasound remission in terms of synovial resident inflammatory cells

**Figure 4:**
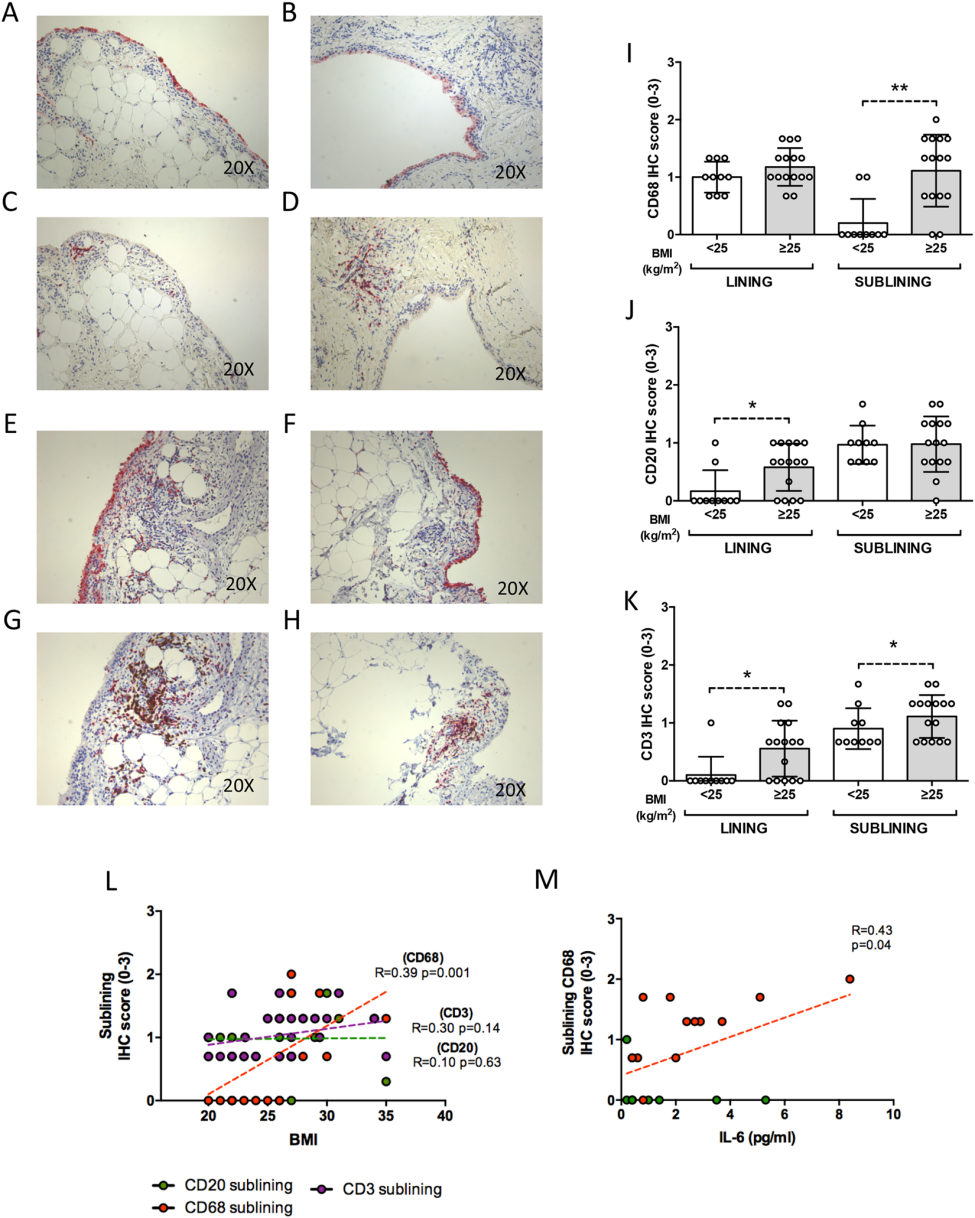
(**A**–**L**) Legend: IHC staining for CD68/CD21 and CD3/CD20 on ST of patients with Rheumatoid Arthritis in clinical and ultrasound remission under MTX + TNF-i based on the BMI category. (**A**,**B**) Example photos of CD68(red)/CD21(brown) staining of ST from RA with BMI < 25 kg/m^2^ in clinical and ultrasound remission under MTX + TNF-i; (**C**,**D**) Example photos of CD3(red)/CD20(brown) staining of ST from RA with BMI < 25 kg/m^2^ in clinical and ultrasound remission under MTX + TNF-i; (**E**,**F**) Example photos of CD68(red)/CD21(brown) staining of ST from RA with BMI ≥ 25 kg/m^2^ in clinical and ultrasound remission under MTX + TNF-i; (**G**,**H**) Example photos of CD3(red)/CD20(brown) staining of ST from RA with BMI ≥ 25 kg/m^2^ in clinical and ultrasound remission under MTX + TNF-i (all magnifications 20X); (**I**) Lining and sublining IHC score for CD68 cells in ST of RA in clinical and ultrasound remission under MTX + TNF-i based on BMI category; Lining and sublining CD68 IHC score of overweight/obese vs normal weight RA in clinical and ultrasound remission under MTX + TNF-i (p = 0.17 and *p < 0.001 respectively); (**J**) Lining and sublining IHC score for CD20 cells in ST of RA in clinical and ultrasound remission under MTX + TNF-i based on BMI category; Lining and sublining CD20 IHC score of overweight/obese vs normal weight RA in clinical and ultrasound remission under MTX + TNF-i (p = 0.95 and *p = 0.02 respectively); (**K**) Lining and sublining IHC score for CD3 cells in ST of RA in clinical and ultrasound remission under MTX + TNF-i based on BMI category; Lining and sublining CD3 IHC score of overweight/obese vs normal weight RA in clinical and ultrasound remission under MTX + TNF-i (*p = 0.02 and *p = 0.04 respectively); (**L**) Correlations between IHC scores of sublining CD68^+^, CD20^+^ and CD3^+^ cells and BMI (R = 0.39, p = 0.001 for CD68^+^, R = 0.10, p = 0.63 for CD20^+^ and R = 0.30, p = 0.14 for CD3^+^ cells respectively) in RA in stable clinical and ultrasound remission under MTX + TNF-i; (**M**) Correlation between IL-6 PB levels and IHC of sublining CD68^+^ cells (R = 0.43; p = 0.04) in RA in stable clinical and ultrasound remission under MTX + TNF-i (red dots indicate RA with BMI ≥ 25 kg/m^2^ and green dots indicate RA with BMI < 25 kg/m^2^, respectively); RA: Rheumatoid Arthritis; IHC: Immunohistochemistry; BMI: Body Mass Index; CD: Cluster designation; MTX: Methotrexate; TNF-i: Tumor Necrosis factor inhibitor.

Among RA in stable clinical and US remission under MTX + TNF-i, patients with BMI ≥ 25 kg/m^2^, showed higher rate of residual synovial inflammation compared to RA with BMI < 25 kg/m^2^ (Fig. 4A–H). In particular, RA in stable clinical and US remission with BMI ≥ 25 kg/m^2^ showed significantly higher IHC score for sublining CD68^+^ (1.2 ± 0.3) (Fig. 4I), lining CD20^+^ cells (0.6 ± 0.4) (Fig. 4J), lining CD3^+^ (0.6 ± 0.5) and sublining CD3^+^ (1.1 ± 0.4) cells (Fig. 4K) compared to RA with BMI < 25 kg/m^2^ (0.2 ± 0.4 for sublining CD68^+^ cells; p < 0.001; 0.2 ± 0.4 for lining CD20^+^ cells; p = 0.02; 0.1 ± 0.3 for lining CD3^+^ cells, p = 0.02; and 0.9 ± 0.3 for sublining CD3^+^ cells; p = 0.04 respectively). In addition, considering the whole cohort of RA in stable clinical and US remission, BMI value directly correlated with the IHC scores for sublining CD68^+^ (R = 0.39; p = 0.001) whereas no correlations were found between BMI and sublining CD3^+^ (R = 0.30; p = 0.14) and CD20^+^ cells (R = 0.10; p = 0.63) (Fig. 4L). Finally, IL-6 plasma levels directly correlated with the IHC score of sublining CD68^+^ cells in ST of RA in stable clinical and ultrasound remission (R = 0.43; p = 0.04) with a higher significance considering RA in sustained remission with BMI ≥ 25 kg/m^2^ (R = 0.66; p = 0.01) (Fig. 4M).

### BMI influences transcriptional signature of synovial tissue of RA patients at disease onset and at the time of sustained remission achievement

**Figure 5:**
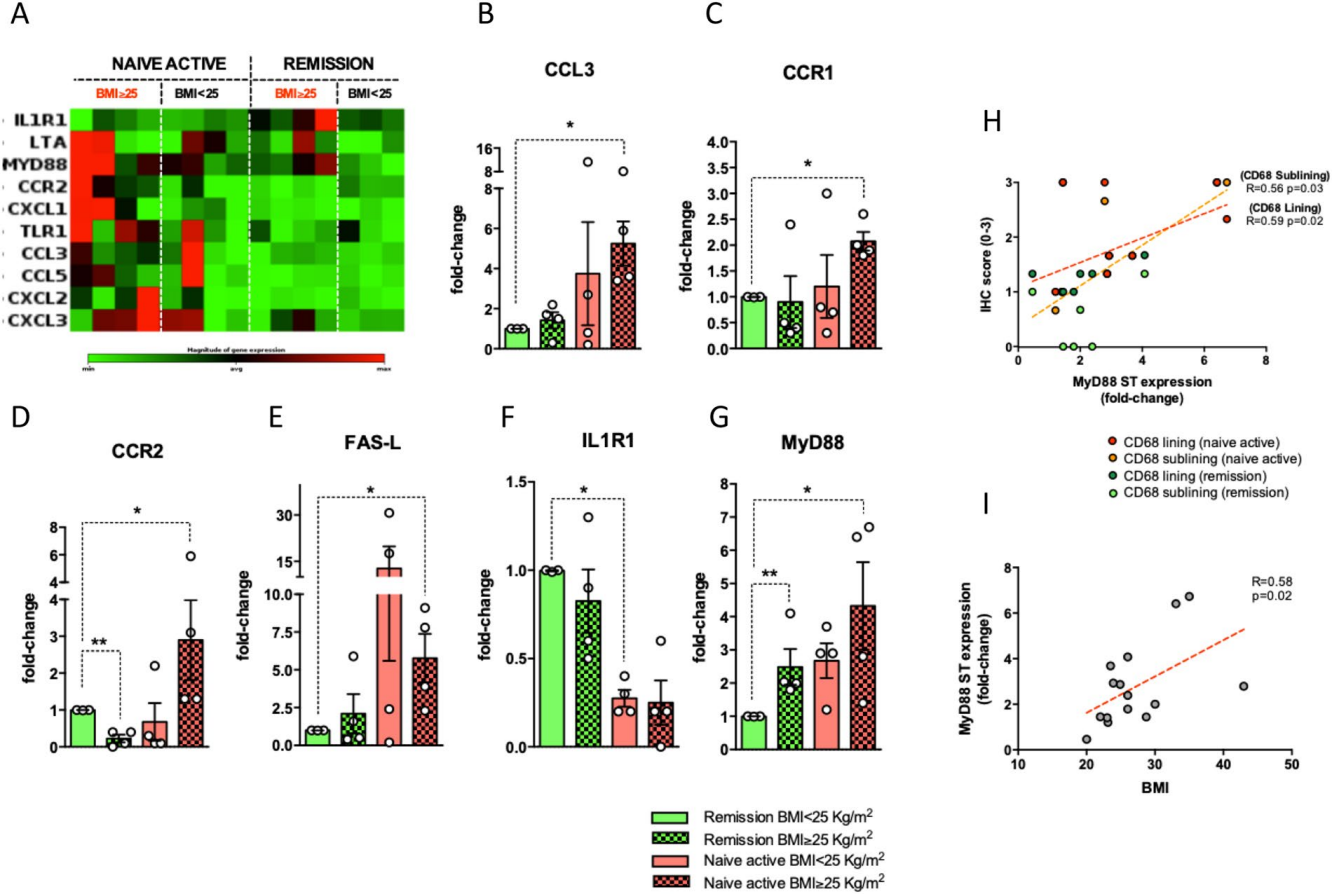
(**A**–**I**) Legend: Gene expression profile of synovial tissue of RA patients with naive to treatment active disease and in sustained clinical and ultrasound remission based on BMI category. (**A**) Clustergram of the dataset displaying a heat map indicating expression of genes across individual samples (naive to treatment active RA and RA in sustained clinical and ultrasound remission based on BMI category); (**B**) CCL3 expression in naive to treatment active RA and RA in sustained clinical and ultrasound remission based on BMI category; *p = 0.02 CCL3 gene expression in synovial tissue of naive to treatment active RA with BMI ≥ 25 Kg/m^2^ compared to RA patients in sustained clinical and ultrasound remission with BMI < 25 Kg/m^2^; (**C**) CCR1 expression in naive to treatment active RA and RA in sustained clinical and ultrasound remission based on BMI category; *p = 0.03 CCR1 gene expression in synovial tissue of naive to treatment active RA with BMI ≥ 25 Kg/m^2^ compared to RA patients in sustained clinical and ultrasound remission with BMI < 25 Kg/m^2^; (**D**) CCR2 expression in naive to treatment active RA and RA in sustained clinical and ultrasound remission based on BMI category; *p = 0.02 CCR2 gene expression in synovial tissue of naive to treatment active RA with BMI ≥ 25 Kg/m^2^ and **p = 0.001 CCR2 in RA patients in sustained clinical and ultrasound remission BMI ≥ 25 Kg/m^2^ compared to RA patients in sustained clinical and ultrasound remission with BMI < 25 Kg/m^2^; (**E**) FAS-L expression in naive to treatment active RA and RA in sustained clinical and ultrasound remission based on BMI category; *p = 0.03 FAS-L gene expression in synovial tissue of naive to treatment active RA with BMI ≥ 25 Kg/m^2^ compared to RA patients in sustained clinical and ultrasound remission with BMI < 25 Kg/m^2^; (**F**) IL1R1 expression in naive to treatment active RA and RA in sustained clinical and ultrasound remission based on BMI category; *p = 0.02 IL1R1 gene expression in synovial tissue of naive to treatment active RA with BMI < 25 Kg/m^2^ compared to RA patients in sustained clinical and ultrasound remission with BMI < 25 Kg/m^2^; (**G**) MyD88 expression in naive to treatment active RA and RA in sustained clinical and ultrasound remission based on BMI category; *p = 0.02 MyD88 gene expression in synovial tissue of naive to treatment active RA with BMI ≥ 25 Kg/m^2^ and **p = 0.04 MyD88 gene expression in synovial tissue of RA in sustained remission with BMI ≥ 25 Kg/m^2^ compared to RA patients in sustained clinical and ultrasound remission with BMI < 25 Kg/m^2^; (**H**) Correlations between MyD88 expression in synovial tissue of RA patients and IHC scores for lining (R = 0.59; p = 0.02) and sublining (R = 0.56; p = 0.03) CD68^+^ cells; (**I**) Correlations between MyD88 expression in synovial tissue of RA patients and BMI value (R = 0.58; p = 0.02); IHC: Immunohistochemistry; CD: Cluster designation. RA: Rheumatoid Arthritis; BMI: Body Mass Index.

Gene expression profile assay (Fig. 5A) revealed that among naive to treatment RA, subjects with BMI ≥ 25 Kg/m^2^ showed significant over-expression of CCL3 (5.23 fold; p = 0.02), CCR1 (2.07 fold; p = 0.03), CCR2 (2.90 fold; p = 0.02), FAS-L (5.77 fold; p = 0.03) and MyD88 (4.33 fold; p = 0.02) compared to RA patients in sustained clinical and ultrasound remission with BMI < 25 Kg/m^2^ (Fig. 5B–G). Interestingly, despite comparable disease control, RA patients in sustained clinical and ultrasound remission with BMI ≥ 25 Kg/m^2^ showed significant over-expression of MyD88 (2.49 fold; p = 0.04) compared to RA patients in sustained clinical and ultrasound remission with BMI < 25 Kg/m^2^ (Fig. 5G). Considering the whole RA cohort used for gene expression profile experiment, synovial tissue expression of MyD88 directly correlates with BMI value (R = 0.58, p = 0.02) and IHC scores for lining and sublining CD68^+^ cells (R = 0.59, p = 0.02 and R = 0.56, p = 0.03 for lining and sublining CD68^+^ cells respectively) in RA patients (Fig. 5H,I).

## Discussion

This study shows that overweight/obesity affects the histological features and the gene expression profile of ST of RA patients at the time of disease onset and at achievement of sustained clinical and ultrasound remission. In addition, overweight/obesity condition at the time of DMARDs beginning influences the rate of disease remission achievement in RA treated according to the T2T strategy regardless to the synovitis pattern found at ST level at disease onset.

RA is a chronic disease in which inflammatory cells aberrantly migrate within the ST contributing to joint inflammation and bone damage^13,14^. Obesity incidence is increasing in the general population^15^, and multiple studies confirmed that obesity is a risk factor associated with RA development^16–18^. Several lines of evidence have proven that adipose tissue is an endocrine organ acting not only on metabolism but even on immune and inflammatory processes by releasing inflammatory molecules promoting the chronicity of the inflammatory response in the target tissue^19^. It has been shown that the overweight/obesity status is associated with a higher degree of systemic inflammation and disease burden in RA patients at disease onset^3,20^, and with an inferior response to treatment^1,6,21–23^. Adipose tissue in normal weight subjects is mostly composed by mature adipocytes, their precursors, fibroblasts, endothelial cells and scarce immune cells, mainly macrophages^24^. During the progressive and excessive fat accumulation occurring in overweight/obese subjects, there is a substantial increase in the number of immune cells and a change in their phenotype towards pro-inflammatory cells contributing to the development of obesity-related local and systemic inflammation (the so called low grade inflammation)^25^. In particular, within the adipose tissue of obese individuals, macrophages are the most abundant resident immune cells characterized by a pro-inflammatory (M1) phenotype, active in the secretion of inflammatory cytokines^10,26^. In the present study, for the first time, we evaluated the histological features of ST of RA patients enrolled at different disease phases (i.e. naive to treatment, inadequately responder to conventional DMARDs and at the time of stable clinical and US remission after MTX + TNF-i combined therapy) stratified according to the BMI category. Considering RA at onset, before any DMARDs treatment, we found that overweight/obese RA showed higher rate of follicular synovitis and higher IHC scores for resident synovial inflammatory cells (i.e. CD68^+^, CD21^+^ and CD20^+^) compared to normal weight ones, with a direct correlation between BMI value and IHC scores for resident inflammatory cells suggesting a tight link between body weight and degree of ST inflammation at RA onset. These findings are in line with Kim *et al*.’s study which investigated the early effect of obesity in RA using preclinical animal models, finding that obese Collagen Induced Arthritis (CIA) mice have an earlier disease onset compared to the lean ones due to a faster immune cells migration within the joint tissue^10^.

To date, despite the direct association between high BMI and disease activity in RA, bone damage is less likely associated with obesity in RA^11,12^, suggesting that protective factors may be present in obese patients. It’s well known that adipose tissue is a major source of IL-1RA^27^, whose release was found to be increased in human obesity^28^. In our cohort, we found that naive overweight/obese RA showed higher IL-1RA plasma levels compared to normal weight RA regardless to the synovitis subtype directly related to IL-6 plasma levels possibly due to the inflammatory status. Moreover, despite we observed no differences at baseline in erosion score comparing overweight/obese and normal weight naïve to treatment RA patients, overweight/obese naive RA without erosive disease showed the lowest IL-6/IL-1RA ratio supporting the concept of an unbalance between pro-inflammatory and osteoclastogenic via OPG/RANK pathway, such as IL-6^29^, and bone protective (as IL-1RA) soluble factors in naive RA based on BMI category. Interestingly, at the time of sustained remission achievement, IL-1RA is significantly higher in overweight/obese RA who did not developed erosive disease, supporting the hypothesis that overweight/obese RA patients may have been protected through IL-1RA pathway from bone damage.

Finally, to investigate the role of overweight/obesity status on ST composition during the remission phase of the disease, we included in the study RA patients in stable sustained clinical and ultrasound remission under combination therapy with MTX + TNF-i as previously described^30^. We found that overweight/obese RA patients reaching stable clinical and ultrasound remission showed higher degree of residual synovitis in terms of synovial CD68^+^, CD20^+^ and CD3^+^ cells whose IHC scores directly correlated with the BMI value at the time of remission achievement. Interestingly, IL-6 plasma levels, despite significantly reduced at the time of remission achievement compared to patients at diagnosis, directly correlates with the IHC score for sublining CD68^+^ cells only in overweight/obese RA in sustained clinical and ultrasound remission, supporting the notion that fat excess can promote IL-6 release, contributing to the persistence of residual synovial inflammation, despite good clinical response. These findings are in line with previous data from animal models showing that obese arthritic mice show a significant delay in remission achievement compared to lean ones due to the aberrant polarization, within the synovial tissue, of macrophages towards a pro-inflammatory (M1) phenotype^10^.

These concepts are strengthened by the findings obtained from the gene expression analysis conducted, in the exploratory cohort, on ST from RA at different disease phases stratified by BMI category, revealing that ST from naive overweight/obese RA is enriched by inflammatory genes as CCL3 and MyD88 compared to normal weight RA in sustained disease remission. Interestingly, ST biopsies from overweight/obese RA in sustained remission showed a persistent over-expression of MyD88 compared to normal weight RA in sustained disease remission. MyD88 (myeloid differentiation primary response gene 88) is a central adaptor molecule for the majority of Toll-like receptors (TLRs), which are the most studied pathogen recognition receptors^31,32^. TLR are trans-membrane receptors that play a crucial role in pathogen recognition and immune response by activating various inflammatory signaling pathways, including MyD88, which then leads to an activation of NF-kB signaling activity^32^. High fat diet was demonstrated to induce an increased expression of TLR in murine adipose tissue causing the activation of MyD88 signaling cascades^33^, and MyD88 expression was found to be increased in both peripheral blood mononuclear cells and subcutaneous adipose tissue of overweight/obese subjects compared to normal weight controls^34^. Moreover, MyD88 deficient mice are partially protected to high fat diet induced obesity^35^. Yu *et al*. demonstrated that MyD88 signaling in myeloid cells participates in the initiation and progression of obesity-induced systemic low grade inflammation since MyD88 deficiency in myeloid cells inhibits macrophage recruitment to adipose tissue and their switch to an M1-like phenotype^36^. These findings are in line with MyD88 expression in ST of RA patients, directly correlating with BMI value regardless to RA phase and with the IHC scores of lining and sublining CD68^+^ cells in ST biopsies supporting the notion that an aberrant persistent over-expression of MyD88, dependent from fat mass excess, is involved in the promotion of residual synovitis persistence in ST of overweight/obese RA, despite sustained disease control.

Therefore, based on our findings we conclude that the early and the resolution phase of RA are influenced by overweight/obesity status in terms of ST inflammation and bone remodelling. Moreover, the ST histological composition found at RA onset strongly supports the lower rate of treatment response, in terms of DAS28 reduction, found in obese patients belonging to early RA cohorts^37^. Therefore, these results suggest that weight control is a crucial aim along the whole disease course in RA and interventional studies, including body weight reduction, are necessary to definitively confirm the biological effect of adipose tissue on the entity of RA inflammation and to envision a personalized approach for obese RA.

## Methods

### Patients recruitment

One hundred and thirty-eight consecutive patients fulfilling the American College of Rheumatology 2010 revised criteria for RA^38^, were enrolled. RA patients were divided into naïve to treatment (n = 70), inadequately responder to Methotrexate (MTX-IR) (n = 43) and patients in stable remission under combination of MTX + TNF-inhibitor (TNF-i) (n = 25). All MTX-IR RA were taking stable dose of MTX (mean dose: 13.8 ± 5.7 mg/week). All RA in sustained clinical (DAS44 < 1.6 for at least 6 months) and ultrasound remission were selected based on the published protocol^29^. For each enrolled RA, clinical and laboratory evaluations included the number of tender and swollen joints on 44, erythrocyte sedimentation rate (ESR), C-reactive protein (CRP) and Disease Activity Score (DAS). Peripheral blood (PB) samples were tested for IgA and IgM-RF (Orgentec Diagnostika, Bouty-UK) and ACPA (Menarini Diagnostics-Italy) using commercial Enzyme-Linked Immunosorbent Assay (ELISA) and ChemiLuminescence Immunoassay (CLIA) methods respectively. At study entry, for each enrolled RA, Body Mass Index (BMI) was assessed^39^, and patients were stratified according to the following cut-off value references: <25 as normal weight; 25–29.9 as overweight; ≥30 as obese, respectively. After study enrolment, all naïve RA started MTX 10 mg/weekly for 2 subsequent administrations and 15 mg/weekly afterwards, according to RA management recommendations^40^. Therefore, each RA was followed every 3 months for at least 12 months in an outpatient setting and DAS value was recorded to assess treatment response and the BMI category was registered. All methods were carried out in accordance with the declaration of Helsinki. All the study experimental protocols were approved by the Ethic Committee of the Università Cattolica del Sacro Cuore (Protocol number: 19526/17) and all subjects provided signed informed consent.

### Immunohistochemistry for CD68^+^, CD21^+^, CD20^+^ and CD3^+^ cells in synovial tissue

All enrolled RA underwent ultrasound guided ST biopsy of the knee following the published protocol^41^. Briefly, sections were stained for CD68 mouse anti-human monoclonal antibody (514H12) (for macrophages) or CD20, mouse anti-human monoclonal antibody (L26) (for B lymphocytes) or CD3 mouse anti-human monoclonal antibody (LN10) (for T lymphocytes) or CD21 mouse anti-human monoclonal antibody (2G9) (for follicular dendritic cells)^42,43^ (all by Leica Biosystem, Newcastle-UK) by immunostainer BOND MAX III (Leica). Double Immunohistochemical staining for CD21/CD68 and CD20/CD3 was performed as previously described^30^. Slides were examined using a light microscope (Leica DM 2000) and classified as diffuse or follicular based on the immunostaining of CD68, CD21, CD20 and CD3 positive cells^42^. Specific lymphoid features of the cellular aggregates were assessed by staining 3–4 μm-thick FFPE consecutive sections for CD3, CD20 and CD21 as previously defined^42,43^. All tissues were evaluated using a numerical score based on the number of positive cells in the lining and sublining areas of the section (three different fields in each section), with a score of 0 indicating no positive cells; 1 indicating <10% positive cells; 2 indicating 10–50% positive cells; and 3 indicating >50% positive cells^30^. Inter-rater agreement coefficients for CD68, CD21, CD20 and CD3 IHC scores were assessed (see Supplementary Table 3).

### ELISA assay for IL-6 and IL-1RA plasma levels evaluation

Naive to treatment RA and RA patients in stable clinical and ultrasound remission were tested for IL-6 and IL-1RA PB levels using commercial Enzyme-Linked ImmunoSorbent Assay (ELISA) kits (all by R&D Systems, United Kingdom). The sensitivity of the test was 0.70 pg/ml for IL-6 and 18.3 pg/ml for IL-1RA respectively.

### Gene expression profile of synovial tissue of RA patients with naive active disease and in sustained clinical and ultrasound remission based on BMI category

Total RNA was isolated from synovial tissue of 15 RA patients (8 from RA with naive active disease and 7 from RA in sustained clinical and ultrasound remission as previously described) using the miRneasy kit (Qiagen). RNA was reverse transcribed using a cDNA conversion kit (Qiagen). The cDNA was used on the real-time RT^2^ Profiler PCR Array (QIAGEN, Cat. no. PAHS-077Z) in combination with RT2 SYBR^®^ Green qPCR Mastermix (Cat. no. 330529). A set of controls was included on each plate which enabled data analysis using ΔΔCt method of relative quantification, assessment of reverse transcription performance and assessment of PCR performance. The RT² Profiler PCR Array enables SYBR Green-based real-time PCR analysis using Biorad iQ5 real-time PCR system as follows: 95 °C for 15 min; 40 cycles of 94 °C for 15 s; 55 °C for 30 s; and 70 °C for 30 s. The relative expression was calculated using the ΔΔCt method (relative gene expression = 2(ΔCt test − ΔCt control)] and is presented in fold increase relative to control. The Web-based GeneGlobe Data Analysis Center was used to analyse the real-time PCR data (Qiagen).

### Statistical analysis

Statistical analysis was performed using SPSS V. 20.0 (SPSS. Chicago, Illinois, USA) and Prism Software (GraphPad, San Diego, California, USA). Categorical and quantitative variables were described as frequencies, percentage and mean ± standard deviation (SD). Data on demographic and clinical features were compared between patients by the non-parametric Mann-Whitney U test or χ^2^ test, as appropriate. Spearman’s rank correlation test was used for correlation in all analyses. For the gene expression profile, data analysis was performed using the supplied software (http://www.qiagen.com/it/shop/genes-and-pathways/data-analysis-center-overview-page/), based on Student’s t-test of the replicate 2^(−ΔCt)^ values for each gene in the tested group and in the control group. Fold-change values > 1 imply an upregulation while fold-change <1 imply down-regulation. using RT^2^ Profiler PCR array, a t-test was used to identify significant differences in gene expression profiles between overweight/obese and normal weight naive active RA, overweight/obese RA in sustained remission and normal weight RA in sustained remission used as control. A value of p ≤ 0.05 was considered statistically significant.

## Supplementary information

Supplementary files
