# Supplementary material for: Overweight/obesity affects histological features and inflammatory gene signature of synovial membrane of Rheumatoid Arthritis
Source: Sci Rep. 2019 Jul 18;9:10420. doi: 10.1038/s41598-019-46927-w (PMC6639364; doi:10.1038/s41598-019-46927-w)
Supplement: Supplementary file 1 — Supplementary files [file 41598_2019_46927_MOESM1_ESM.docx]

***Supplementary Material***

**Overweight/obesity affects histological features and inflammatory gene signature of synovial membrane of Rheumatoid Arthritis.**

**Stefano Alivernini^1,2^^, Barbara Tolusso^1^^, Maria Rita Gigante^1^, Luca Petricca^1^, Laura Bui^3^, Anna Laura Fedele^1^, Clara Di Mario^2^, Roberta Benvenuto^3^, Francesco Federico^3,4^, Gianfranco Ferraccioli^2^ and Elisa Gremese*^1,2^.**

^1^Division of Rheumatology, Fondazione Policlinico Universitario A. Gemelli IRCCS, Rome, Italy.

^2^Institute of Rheumatology, Università Cattolica del Sacro Cuore, Rome, Italy.

^3^Institute of Pathology, Fondazione Policlinico Universitario A. Gemelli IRCCS, Rome, Italy.

^4^Institute of Pathology, Università Cattolica del Sacro Cuore, Rome, Italy.

***Corresponding Author**:

Elisa Gremese MD

Division of Rheumatology

Fondazione Policlinico Universitario A. Gemelli IRCCS - Università Cattolica del Sacro Cuore

Via Giuseppe Moscati, 31, 00168, Rome, Italy.

email address: elisa.gremese@unicatt.it

**Supplementary Table 1. Demographic, clinical and immunological characteristics of the study cohorts based on BMI category**

|  | **Naive RA**  **(n=70)** | | ***p*** | **MTX-IR RA**  **(n=43)** | | ***p*** | **Remission RA**  **(n=25)** | | ***p*** |
| --- | --- | --- | --- | --- | --- | --- | --- | --- | --- |
|  | **BMI<25**  **(28)** | **BMI>25**  **(n=42)** |  | **BMI<25**  **(n=18)** | **BMI>25**  **(n=25)** |  | **BMI<25**  **(n=10)** | **BMI>25**  **(n=15)** |  |
| **Female, n(%)** | 22 (78.6) | 33 (78.6) | *1.00* | 16 (88.9) | 23 (92.0) | *0.73* | 9 (90.0) | 12 (80.0) | *0.63* |
| **Age, years** | 48.8±16.4 | 58.6±13.7 | ***0.01*** | 53. 1±17.5 | 64.1±7.9 | *0.04* | 55.5±13.4 | 58.3±16.3 | *0.57* |
| **Disease duration, years** | 1.2±1.2 | 1.8±1.6 | *0.81* | 7.0±5.3 | 5.5±4.0 | *0.42* | 8.2±4.5 | 10.8±7.2 | *0.46* |
| **AB positivity, n(%)** | 14 (50.0) | 22 (52.4) | *1.00* | 9 (50.0) | 16 (64.0) | *0.35* | 7 (70.0) | 10 (66.7) | *1.00* |
| **DAS value** | 3.9±1.2 | 3.5±1.2 | *0.22* | 3.3±1.0 | 3.3±0.9 | *0.97* | 1.1±0.5 | 1.1±0.4 | *0.81* |
| **ESR, mm/1^st^ hour** | 54.9±30.0 | 51.4±28.6 | *0.34* | 50.3±38.9 | 47.1±28.1 | *0.83* | 16.2±11.8 | 17.5±18.8 | *0.72* |
| **CRP, mg/L** | 22.1±21.9 | 22.0±22.5 | *0.89* | 18.9±26.4 | 15.6±19.0 | *0.86* | 1.4±0.9 | 2.6±2.4 | *0.16* |
| **Treatment regimen** |  |  |  |  |  |  |  |  |  |
| **MTX dose, mg/w** | - | - | - | 13.1±5.2 | 14.0±6.1 | *0.76* | 14.0±3.3 | 14.7±4.0 | *0.81* |
| **Etanercept 50 mg/w** | - | - | - | - | - | - | 5 (50.0) | 8 (53.3) | *0.87* |
| **Adalimumab 40 mg/2w** | - | - | - | - | - | - | 5 (50.0) | 7 (46.7) | *0.87* |

**Supplementary Table 1. Demographic, clinical and immunological characteristics of the study cohorts stratified based on BMI category. AB**: autoantibody; **BMI**: Body Mass Index; **CRP**: C Reactive Protein; **DAS**: Disease Activity Score; **ESR**: Erythrocyte Sedimentation Rate; **RA**: Rheumatoid Arthritis. **MTX-IR**: Methotrexate inadequate responder; **SD**: Standard Deviation; **w**: week; Data are expressed as mean ± SD or number(percentage); **Bold**: p<0.05.

**Supplementary Figure 1 (A-C): IL-6 and IL-1RA plasma levels in RA patients naive to treatment and at remission achievement stratified based on erosive disease.**

**Supplementary Figure 1 Legend: (A)** IL-6 plasma levels in naive and remission RA based on the presence of erosive disease (38.4 ± 54.3 pg/ml vs 66.6 ± 56.9 pg/ml in naive RA without and with erosive disease respectively, *p=0.05; 3.5 ± 2.0 pg/ml vs 3.9 ± 2.6 pg/ml in RA in remission without and with erosive disease respectively, p=0.72); **(B)** IL-1RA plasma levels in naive and remission RA based on the presence of erosive disease (571.1 ± 374.1 pg/ml vs 789.5 ± 412.0 pg/ml in naive RA without and with erosive disease respectively, *p=0.04; 577.0 ± 221.0 pg/ml vs 316.9 ± 88.9 pg/ml in RA in sustained remission without and with erosive disease respectively, **p=0.002); (C) Plasma IL-6/IL-1RA ratio in naive to treatment RA patients stratified based on BMI category and erosive disease, *p=0.01 normal weight naive RA with erosive disease (0.13 ± 0.05) vs overweight/obese naive RA without erosive disease (0.03 ± 0.01); **p=0.01 normal weight naive RA without erosive disease (0.13 ± 0.04) vs overweight/obese naive RA without erosive disease (0.03 ± 0.01); ***p=0.01 overweight/obese naive RA with erosive disease (0.09 ± 0.08) vs overweight/obese naive RA without erosive disease (0.03 ± 0.01).

**Supplementary Table 2: Correlations between IL-1RA plasma levels, IL-6 plasma levels and BMI value in RA patients naive to treatment and at remission achievement.**

|  | **Naive RA** | | **Remission RA** | |
| --- | --- | --- | --- | --- |
|  | **PB IL-1RA, pg/ml** | **PB IL-6, pg/ml** | **PB IL-1RA, pg/ml** | **PB IL-6, pg/ml** |
| **PB IL-6, pg/ml** | **R=0.38**  **p=0.003** | - | R=0.10  p=0.71 | - |
| **PB IL-1RA, pg/ml** | - | **R=0.38**  **p=0.003** | - | R=0.10  p=0.71 |
| **BMI, Kg/m^2^** | **R=0.35**  **p=0.01** | R=-0.01  p=0.93 | **R=0.44**  **p=0.03** | **R=0.41**  **p=0.05** |

**RA**: Rheumatoid Arthritis; **PB**: peripheral blood; **IL**: Interleukin; **BMI**: Body Mass Index; **Bold** indicates p values <0.05.

**Supplementary Figure 2(A,B): Rate of DAS remission achievement in RA patients treated according to the treat to target strategy based on BMI category and synovitis pattern.**

**Supplementary Figure 2 Legend: (A)** Percentages of DAS-remission achievement in RA patients treated with the treat to target strategy at 6 and 12 months follow-up, based on the BMI category; RA patients with BMI < 25 kg/m^2^ vs RA patients with BMI ≥ 25 kg/m^2^ at 6 months follow-up, *p=0.02; RA patients with BMI < 25 kg/m^2^ vs RA patients with BMI ≥ 25 kg/m^2^ at 12 months follow-up, *p=0.01; **(B)** Percentages of DAS-remission achievement in RA patients treated with the treat to target strategy at 6 and 12 months follow-up, based on the BMI category and synovitis pattern; RA patients with BMI < 25 kg/m^2^ and diffuse synovitis vs RA patients with BMI ≥ 25 kg/m^2^ and diffuse synovitis, at 6 months follow-up, *p=0.004; RA patients with BMI < 25 kg/m^2^ and diffuse synovitis vs RA patients with BMI ≥ 25 kg/m^2^ and follicular synovitis, at 6 months follow-up, **p=0.005; RA patients with BMI < 25 kg/m^2^ and diffuse synovitis vs RA patients with BMI ≥ 25 kg/m^2^ and follicular synovitis, at 12 months follow-up, ***p=0.03; **RA**: Rheumatoid Arthritis; **DAS**: Disease activity Score; **BMI**: Body mass Index.

**Supplementary Table 3.** Inter-rater agreement coefficients for CD68, CD21, CD20 and CD3 IHC scores.

|  | **Inter-rater coefficient*** |
| --- | --- |
| **Lining CD68 IHC score** | R=0.64; p<0.001 |
| **Sublining CD68 IHC score** | R=0.54; p=0.01 |
| **Lining CD21 IHC score** | R=0.73; p<0.001 |
| **Sublining CD21 IHC score** | R=0.81; p<0.001 |
| **Lining CD20 IHC score** | R=0.55; p<0.001 |
| **Sublining CD20 IHC score** | R=0.68; p<0.001 |
| **Lining CD3 IHC score** | R=0.56; p<0.001 |
| **Sublining CD3 IHC score** | R=0.65; p<0.001 |

**IHC**: Immunohistochemistry. *Pearson Correlation coefficient.
